# Supplementary material for: Valine-279 Deletion–Mutation on Arginine Vasopressin Receptor 2 Causes Obstruction in G-Protein Binding Site: A Clinical Nephrogenic Diabetes Insipidus Case and Its Sub-Molecular Pathogenic Analysis
Source: Biomedicines. 2021 Mar 15;9(3):301. doi: 10.3390/biomedicines9030301 (PMC8002004; doi:10.3390/biomedicines9030301)
Supplement: Supplementary file 1 [file biomedicines-09-00301-s001.pdf]

Table S1. Primers of human *AVPR2* and *AQP2* used for Sanger sequencing.

Primer	Sequence	Size (bp)
hAQP2-1F	AAAGAGAGCGATAGAGTGCG	475
hAQP2-1R	CCTTGTGGATGGCAAAGTTG	
hAQP2-2F	GAAGAAGGGATCAGTCGTTG	475
hAQP2-2R	TGGGGTATCTAGGAGTCAAAC	
hAQP2-3F	TCAAGAGGAACAGACACCC	497
hAQP2-3R	AAGCTGTTGTCATCCTCATTC	
hAQP2-4F	TAACCCCGCACTGACAAGG	522
hAQP2-4R	ACGTCCAGGAAGCAGCTAC	
hAVPR2-1F	TGACCATCCCTCTCAATCTTC	
hAVPR2-1R	CTCCAGGAAAGCCATGTTATG	474
hAVPR2-2.1F	AACATGGCTTTCCTGGAGTCC	
hAVPR2-2.1R	AGGCATACATGCCCACCATC	483
hAVPR2-2.2F	CCCATACACGTCTTCATTGGC	
hAVPR2-2.2R	GCACCAGACTGGCATGAATC	496
hAVPR2-2.3F	ACCTATGTCACCTGGATTGCCC	
hAVPR2-2.3R	ACACGCTGCTGCTGAAAGATGC	494
hAVPR2-3F	ACCCAACCTAGATCCTCCAC	
hAVPR2-3R	CTGAAGCTCTCCTCATACAGC	436

Table S2. All simulation systems parameters.

Simulation System	Simulation Time (μs)	Total Number of Atoms	Number of Sodium Ions	Number of Chloride Ions	Number of Waters	Number of Lipids
AVPR2-WT	1.5 ×2	79588	92	101	21294	240
AVPR2-D136A	1.5 ×2	78578	92	102	20958	240
AVPR2-ΔV279	1.5 ×2	78266	92	101	20856	240
